# Supplementary material for: Green Synthesis of Molecularly Imprinted Polymers for Dispersive Magnetic Solid-Phase Extraction of Erythrosine B Associated with Smartphone Detection in Food Samples
Source: Materials (Basel). 2022 Oct 31;15(21):7653. doi: 10.3390/ma15217653 (PMC9655850; doi:10.3390/ma15217653)
Supplement: Supplementary file 1 [file materials-15-07653-s001.zip › materials-1974903-supplementary.pdf]

materials

Supplementary Material

Green synthesis of molecularly imprinted polymers for dispersive magnetic solid phase extraction of erythrosine B associated with smartphone detection in food samples

Dounia Elfadil [1,2], Flavio Della Pelle [1], Dario Compagnone [1,*] and Aziz Amine [2,*]

[1] Faculty of Bioscience and Technology for Food, Agriculture and Environment, University of Teramo, via Renato Balzarini 1, 64100 Teramo, Italy
[2] Laboratory of Process Engineering and Environment, Faculty of Sciences and Techniques, Hassan II University of Casablanca, Mohammedia 20650 Morocco
[*] Correspondence: dcompagnone@unite.it (D.C.); aziz.amine@fstm.ac.ma (A.A.)

Table S1. Equations employed for isotherm and kinetic studies.

Model	Equation	Parameters
Langmuir	$Q_e = \frac{Q_{max}K_LC_e}{1+C_e}$	k_L (L/mg) is the Langmuir constant; Q_{max} (ng/mg) represents the maximum adsorption capacity obtained with Langmuir model.
Freundlich	$Q_e = K_FC_e^{1/n}$	k_F (mg/g) and n_F are the Freundlich model constants.
Pseudo-first order	$Q_t = Q_e(1-e^{-k_1t})$	Q_t (mg·g^{-1}) is the adsorption capacity at different time intervals, t (min) is the adsorption time and $k_{1,}$ (min^{-1}) and k_2 (g·mg·min^{-1}) are the rate constants of the PFO and PSO models, respectively
Pseudo-second order	$Q_t = \frac{k_2{Q_e}^2t}{1+k_2Q_et}$	

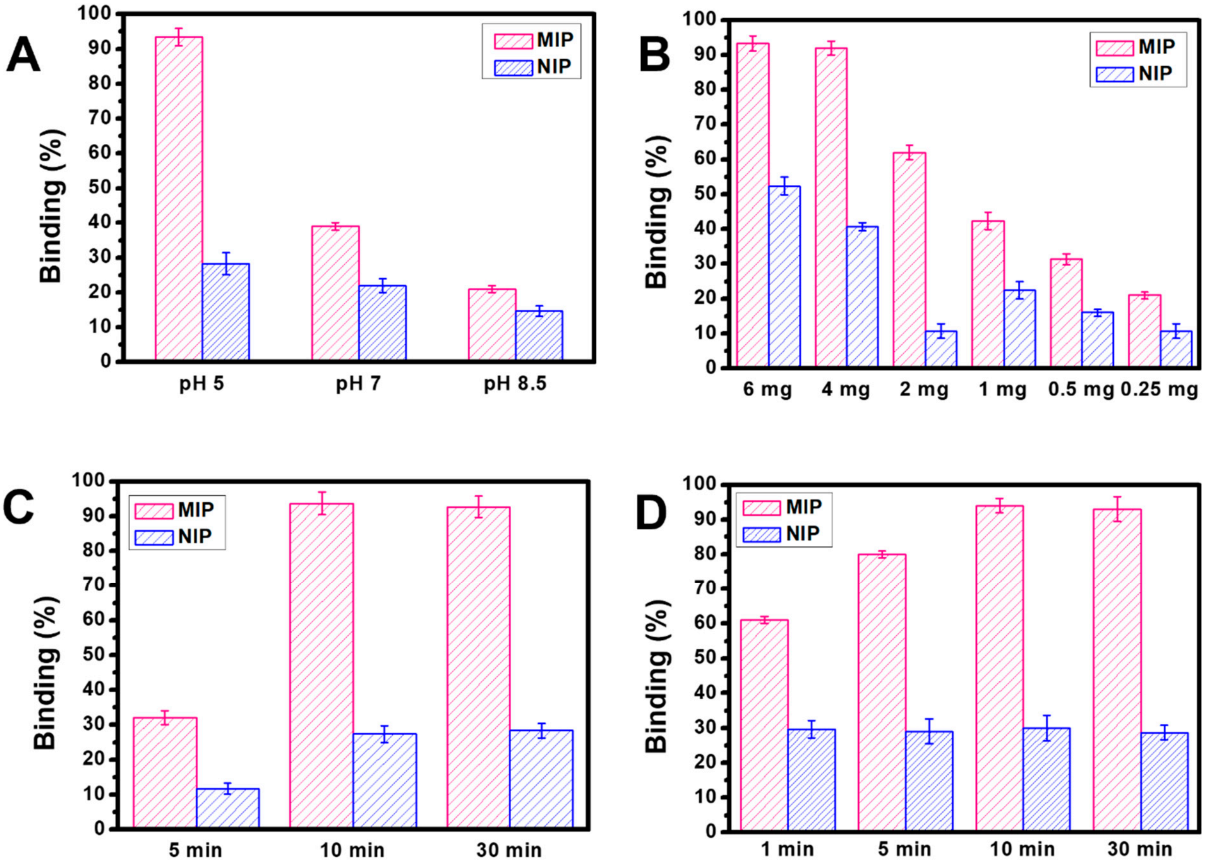

Figure S1. Optimization of MIP-MSPE. (**A**) effect of pH using 4 mg of imprinted polymer, binding time 30 min; (**B**) Effect of the amount of imprinted polymer in acetate buffer (pH 5), binding time 30 min; (**C**) effect of time in the adsorption step using 4 mg of imprinted polymer in acetate buffer (pH 5); (**D**) effect of time in the elution step using 4 mg of imprinted polymer, using 0. 1 M NaOH as elution solution.

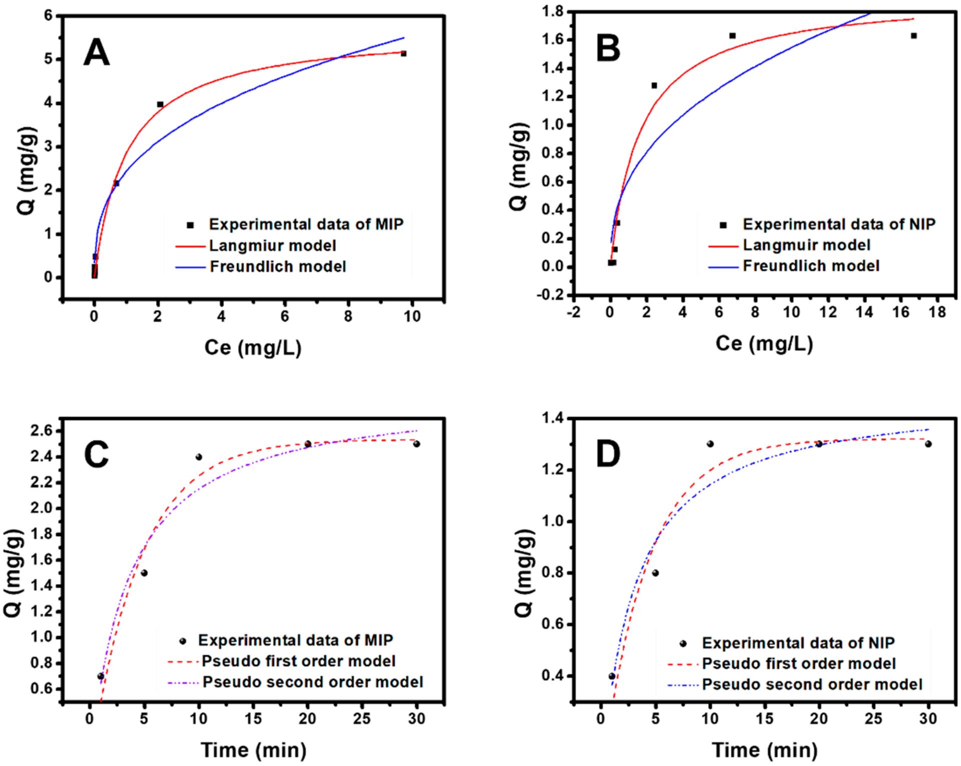

Figure S2. Experimental data for ERT-B adsorption by (**A**) MIP and (**B**) NIP at different concentrations;,in each graph is reported the adsorption isotherm obtained with the Langmuir and Freundlich models. Kinetic adsorption experimental data obtained for ERT-B onto (**C**) MIP and (**D**) NIP; in each graph are reported the obtained adsorption of non-linear pseudo first order and pseudo second order models.

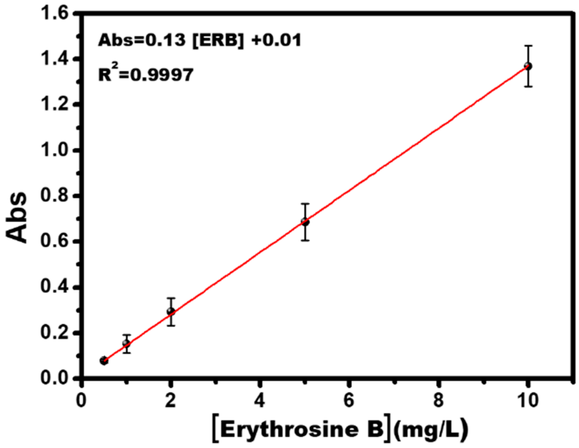

Figure S3. Calibration curve for ERT-B determination using spectrophotometric method.

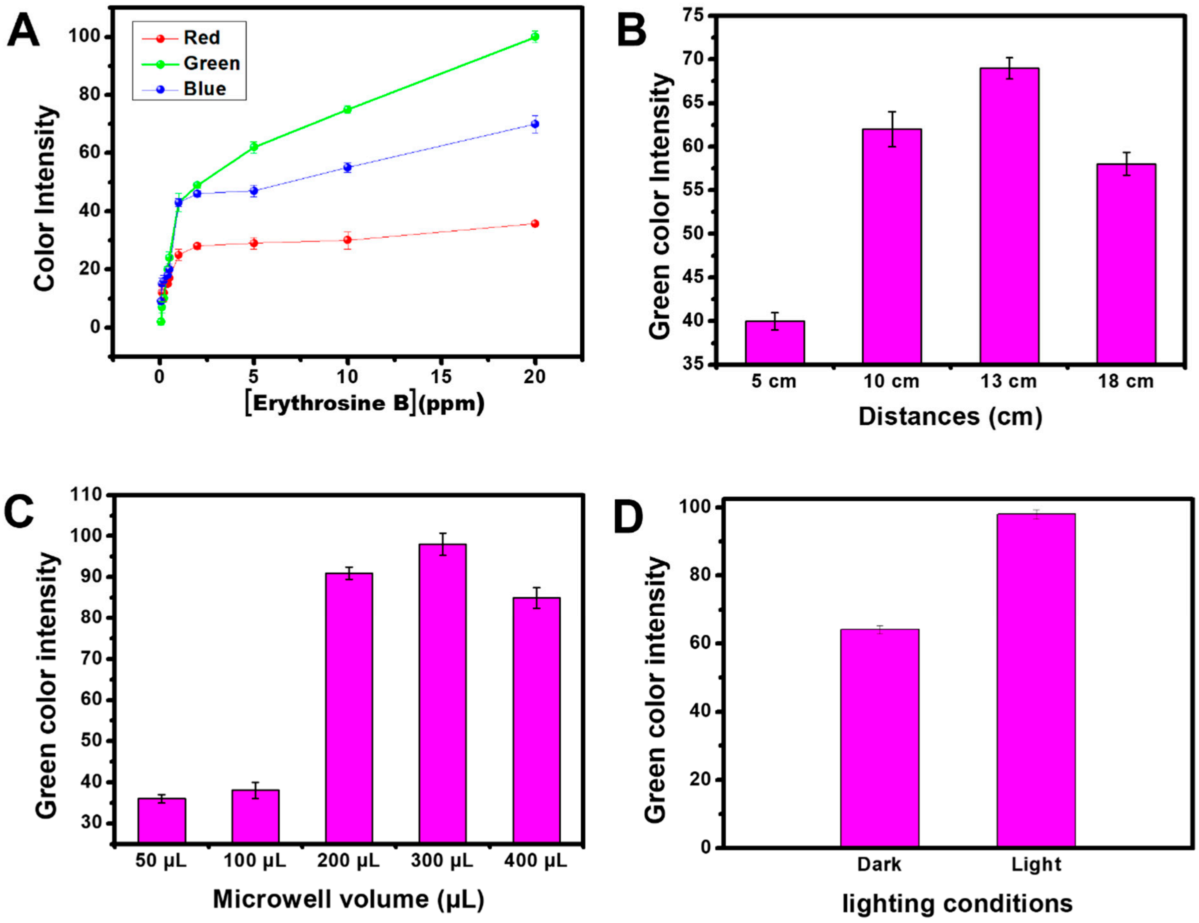

Figure S4. (**A**) Relationship between the intensity of red, green and blue color values for ERT-B in the range of 0.05 to 20 ppm; (**B**) freen color intensities obtained as function of different focal distances between the microwell and smartphone camera; (**C**) green color intensities obtained as function of different volume of ERT-B solution in the microwell; (**D**) Green color intensities obtained in dark (flashlights on) and in light. Data are reported as mean ± SD, n = 3.

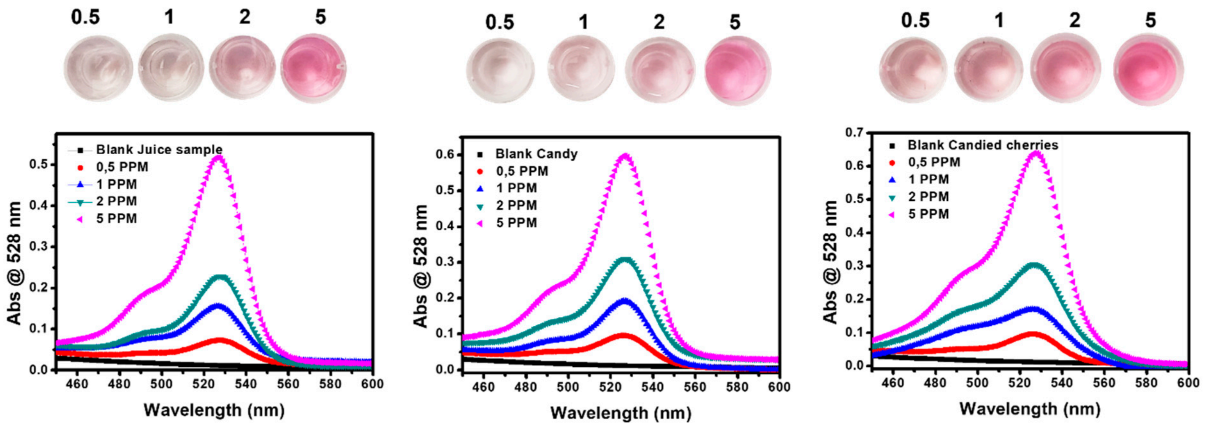

(**A**) (**B**) (**C**)

Figure S5. Pictures and spectra of the fortified samples extracted using the MIP-MDSPE procedure. (**A**) spiked juice, (**B**) candy and (**C**) candied cherries.
